# The role of complement in ATTR amyloidosis: a new therapeutic avenue?

**DOI:** 10.1186/1750-1172-10-S1-P3

**Published:** 2015-11-02

**Authors:** Elena Panayiotou, Eleni Fella, Rebecca Papacharalambous, Stavros Malas, Theodore Kyriakides

**Affiliations:** 1 Cyprus Insitute of Neurology and Genetics, Non-profit Private Academic, Medical Center, 2370, Nicosia, Cyprus

## Background

Familial Amyloidotic Polyneuropathy Type I is a lethal autosomal dominant sensorimotor and autonomic neuropathy due to deposition of amyloid fibrils composed of aberrant transthyretin (TTR) protein (ATTR neuropathy). A substitution of valine for methionine at position 30 of the protein is the commonest mutation. ATTRMet30 neuropathy exhibits a great degree of variability, both in the age of onset as well as penetrance among different populations. The penetrance in Cyprus is 28% compared to 2% and 80% in north Sweden and Portugal respectively. Genetic and epigenetic factors have been implicated and although we have previously demonstrated a correlation of complement C1q polymorphisms with age of onset among the Cypriot population, the exact mechanisms remain undetermined. The complement cascade, as a whole, has long been investigated for its association with inflammation and macromolecule aggregate clean-up. In the mouse model of Alzheimer disease, C1q has been shown to modulate beta-amyloid induced complement activation and neuronal loss. C1q has also been shown to be neuroprotective against toxic concentrations of serum amyloid P and to modulate phagocytosis of soluble pre-amyloid aggregates. Thus C1q appears to be strong candidate for being a modifier in the phenotype of ATTRMet30 neuropathy.

## Methods

A transgenic mouse model of ATTRMet30 was cross bred with a C1q knockout strain in order to produce a complement deficient ATTRMet30 strain. In addition, the C5a receptor inhibitor PMX53 was administered to the original ATTRMet30 mouse model. Conventional and real-time PCR were carried out to characterize all mice, Thioflavin S, immunocytochemistry and immunoblotting were utilized to assess amyloid and a number of molecular markers of apoptosis, oxidative stress and endoplasmic reticulum stress.

## Results

Amyloid deposition was increased by over 30% by C1q ablation and by over 600% by PMX53 administration. A parallel increase was also recorded in apoptotic and pathogenic markers such as MMP9, BiP (GRP78), Fas and Caspase-3.

## Conclusion

Whereas the exact role of complement in FAP has not yet been fully elucidated, it is likely that localized activation of certain complement components may contribute to the successful removal of amyloid. Complement manipulation can perhaps be potentially exploited therapeutically as a generic therapy in amyloidosis.

